# *Diacamma* ants adjust liquid foraging strategies in response to biophysical constraints

**DOI:** 10.1098/rspb.2023.0549

**Published:** 2023-06-14

**Authors:** Haruna Fujioka, Manon Marchand, Adria C. LeBoeuf

**Affiliations:** ^1^ Faculty of Environmental and Life Science, Okayama University, Okayama, Japan; ^2^ Department of Biology, University of Fribourg, Fribourg, Switzerland; ^3^ Department of Physics, University of Fribourg, Fribourg, Switzerland

**Keywords:** mandibular pseudotrophallaxis, social bucket, liquid transportation, liquid collection, optimal foraging theory, biophysics

## Abstract

Ant foragers provide food to the rest of the colony, often requiring transport over long distances. Foraging for liquid is challenging because it is difficult to transport and share. Many social insects store liquids inside the crop to transport them to the nest, and then regurgitate to distribute to nest-mates through a behaviour called trophallaxis. Some ants instead transport fluids with a riskier behaviour called pseudotrophallaxis—holding a drop of liquid between the mandibles through surface tension. Ants share this droplet with nest-mates without ingestion or regurgitation. We hypothesised that ants optimize their liquid-collection approach depending on viscosity. Using an ant that employs both trophallaxis and pseudotrophallaxis, we investigated the conditions where each liquid-collection behaviour is favoured by measuring biophysical properties, collection time and reaction to food quality for typical and viscosity-altered sucrose solutions. We found that ants collected more liquid per unit time by mandibular grabbing than by drinking. At high viscosities ants switched liquid collection method to mandibular grabbing in response to viscosity and not to sweetness. Our results demonstrate that ants change transport and sharing methods according to viscosity–a natural proxy for sugar concentration–thus increasing the mass of sugar returned to the nest per trip.

## 1. Introduction

Efficient foraging is crucial for animals to survive, grow and reproduce. Organisms need to balance energy spent with energy gained [1,2]. Optimal foraging theory assumes that animals' foraging decision making has evolved to the point that the fitness of individuals has been maximized. Foraging provides energy to survive and reproduce; however, it has the cost of exposing the individual to being preyed upon by other animals and it costs energy, for example, due to the time spent exploiting, processing and transporting food.

Animals have a wide variety of foraging strategies. Ants are central-place foragers with diverse diets ranging from complete herbivory to complete predation [3–6]. Morphological and phylogenetic evidence suggests that the ancestral ant was a predator, and transitions to herbivory occurred several times in predatory lineages [5,7,8]. Plant-based food sources, such as plant nectar [9] or honeydew excreted by sapsucking Hemiptera and scale insects [10–12], are rich in carbohydrates. In many species and especially in ecologically dominant ant lineages, these sugary liquids are ants’ main source of energy [13]. Additionally, liquid resources, such as honeydew or nectar, are less ephemeral relative to insect prey and incur fewer risks for ant foragers relative to hunting. Thus, the use of plant-based food sources may lead to lower foraging time and less risk for foragers per calorie returned to the nest.

Transportation of liquid food is a foraging challenge. Many ants and bees transport liquid stored inside their crop, where it cannot easily be lost or stolen [14] during transport. Foragers regurgitate this fluid to distribute it to nest-mates through a behaviour called trophallaxis. Many liquid-feeding ants have acquired morphological adaptations for trophallaxis. The ant crop is separated from their midgut by a variably developed proventriculus, which allows the crop to store a large amount of liquid in some species [15]. The structure of the proventriculus varies considerably across taxa [16–18], and liquid-feeding ants often have a more elaborate proventriculus. The gaster and crop also need to be expandable to best store liquid food, either temporarily for transport or over the long term in the case of repletes. The extreme example of morphological specialization are honeypot ants *Myrmecocystus* (Formicinae), where replete workers have a massive ball-like distended gaster full of food to the point where they can barely move [19]. Such species rely on trophallaxis to redistribute food from the repletes to the rest of the colony. Although trophallaxis is considered a safe and reliable liquid transportation method for ants, the crop load (i.e. liquid food intake) strongly depends on these morphological constraints.

Some ants do not have these morphological specializations but nonetheless consume liquid food: Ectatomminae (*Ectatomma*), Ponerinae (*Diacamma, Neoponera, Odontomachus, Paraponera, Pachycondyla, Rhytidoponera*). These ants typically use mandibular pseudotrophallaxis (hereafter called pseudotrophallaxis) as their method of liquid transport [15,20]. Instead of storing liquid inside the crop, foragers hold liquid food between their mandibles where it forms a droplet maintaining its round shape through surface tension. After foragers return to the nest, they pass the liquid food to nest-mates without regurgitation. In nature, ants use this method to collect and transport water, honeydew, plant fluids such as nectar and animal fluids such as haemolymph [21,22]. Previous studies in ponerine ants have reported how this behaviour allows liquids to be distributed in the nest [23,24]. This liquid transport method is sometimes referred to as the ‘social bucket’ method and has been suggested to be an evolutionary precursor to ‘true’ trophallaxis [7].

Handling time is crucial for efficient foraging. For liquid food, the handling time includes both the speed of food collection (i.e. drinking time or grabbing time) and the transport time to the nest. Drinking time in ants has been shown to depend on food quality such as sugar concentration and viscosity itself [25]. Previous studies found that drinking time increased linearly with increasing sugar concentration [25–29]. Individuals need to decide when to stop drinking, considering the balance between energy gain and predation risk. The handling time of pseudotrophallaxis has not been investigated. Regarding transport success, once foragers store food in the crop, they can transport the liquid food safely back to the nest. When using pseudotrophallaxis, there is the possibility of losing the liquid food along the return path. Also, keeping mandibles open adequately may increase the likelihood of predation.

Some ants use both behaviours. The ponerine ant *Diacamma* cf. *indicum* from Japan performs both trophallaxis and pseudotrophallaxis [30], and has a simple proventricular morphology and a rigid, non-extensible gaster. Thus, *Diacamma* cf. *indicum* is an ideal model species to investigate efficient foraging strategies regarding liquid food because it allows us to investigate foraging strategies without morphological specialization.

The aim of the present study is to reveal what leads ants to use the collection mode of mandibular grabbing instead of drinking and whether ants' liquid collection modes maximize calorie intake rates per foraging trip. We hypothesize that viscosity triggers a switch in collection behaviour between drinking and mandibular grabbing, where mandibular grabbing is more efficient in collecting high-viscosity solutions. To test this hypothesis, we conducted experiments to investigate 1) physical properties of the liquids such as capillary length, viscosity and surface tension; 2) drinking intake rate; 3) volume and time of liquid food collection depending on sugar concentration; 4) whether ants change their transportation method depending on sugar concentration or viscosity by dissociating viscosity from sugar concentration; and 5) the foraging efficiency for each approach using walking speed and the success rate of mandibular transport to estimate the total amount of sugar carried per trip.

## 2. Results

**Figure 1.**
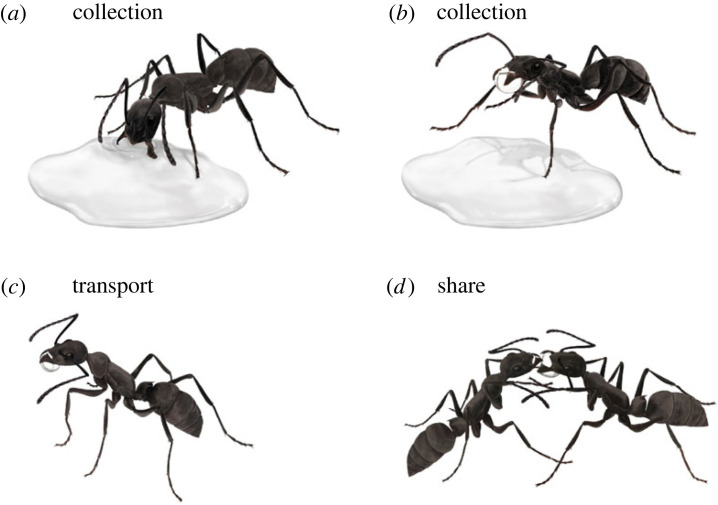
Ethogram of the social bucket technique. An ant touches a solution with the antennae and mouthparts (tasting). After tasting, an individual opens mandibles to grab and pull at the liquid (*a*). The ant occasionally succeeds in collecting a droplet of solution between the mandibles (*b*). The individual returns to the nest (*c*). Inside the nest, the ant shares the drop with other nest-mates (*d*). When the receiver begs for the droplet, their antennae move quickly. Several nest-mates can drink from the droplet at the same time. Illustrations by Ken Naganawa.

*Diacamma* ants forage for sugary liquids and use two forms of behaviour to collect liquid—drinking and mandibular grabbing (figure 1). To understand whether ants alter their liquid collection and transport behaviour according to the contents and properties of the liquid they are transporting, we measured multiple variables: biophysical properties of the liquids, drinking intake rate, volume carried, time spent collecting, frequencies of different foraging actions, sugar load acquired per trip, walking speed and success rate of transport.

**Figure 2.**
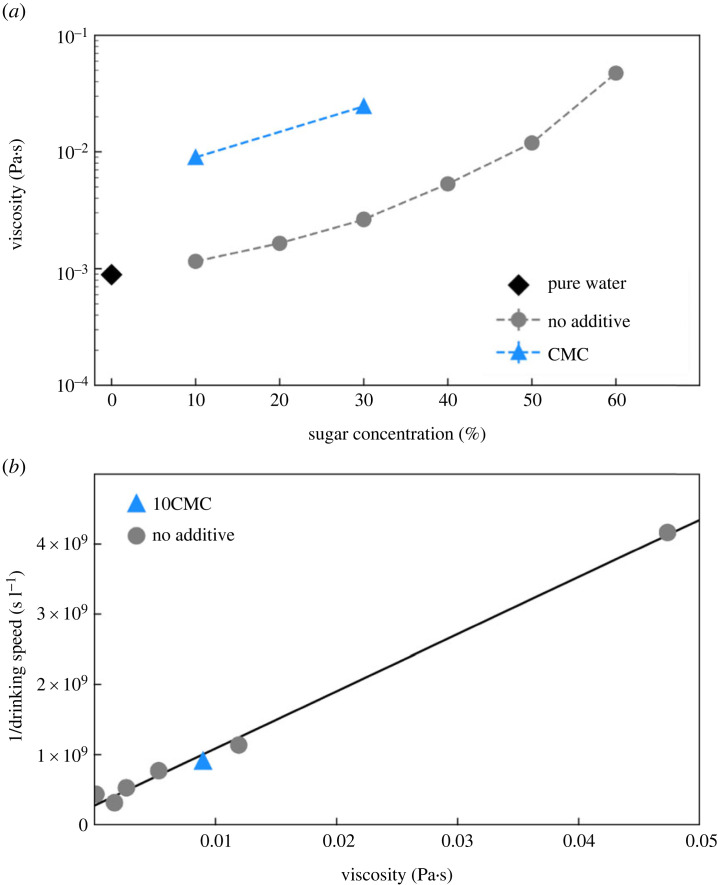
Dynamic viscosity dictates drinking intake rate. (*a*) Dynamic viscosities at 25°C for different sugar water solutions as a function of the sugar concentration (w/w). The different colours correspond to water (circles and diamond) and viscosity-altered solutions (triangles). The error bars (which are barely visible because of their small value) denote the quality of the Newtonian fit applied to each flow curve for each solution. The blue triangles indicate the viscosity-altered solutions 10CMC and 30CMC sugar solution with 0.25 CMC (w/w). (*b*) The relationship between drinking intake rate and viscosity. The inverse of drinking intake rate increases linearly with dynamic viscosity for sugar solutions with or without viscosity-altering additives. CMC, carboxymethylcellulose sodium salt.

First, we analysed three liquid properties of a range of ecologically relevant sugary liquids from 0–60% sucrose [31]: dynamic viscosity, surface tension and capillary length. Dynamic viscosity increased with increasing sugar water concentration (figure 2*a*). We altered the viscosity of a low-sugar solution using the viscosity-modifying additive carboxymethylcellulose sodium salt (CMC), a non-toxic inert viscosity modifier [32]. The viscosity of 10% sugar water with CMC (10CMC) was comparable to the viscosity of 40–50% sugar water (electronic supplementary material, table S1, figure 2*a*). Surface tension is the energy it costs to create new unit surface area of a liquid. Capillary length is the size above which a drop of liquid can no longer sustain its own shape by surface tension, but starts to be deformed and to flow under its own weight. We measured an increase in surface tension (mN m^−1^) with increasing sugar concentration while the capillary length was not significantly affected (table 1).

**Table 1.** Surface tension and capillary length for water/sugar solutions.

sugar concentration (%)	surface tension (mN m^−1^)	std. error surface tension (mN m^−1^)	density (g ml^−1^)	capillary length (mm)
0	71.7	0.1	0.9982	2.71
10	70.7	0.5	1.022	2.66
20	74.7	0.4	1.0631	2.68
30	80.0	0.4	1.1132	2.70
40	77.8	0.3	1.1374	2.64
50	78.8	0.3	1.1975	2.59
60	84.5	0.3	1.2391	2.64

Combining video recording and weight measurements of ants before and after drinking, we determined that drinking intake rate (volumetric flow rate, in µl s^−1^) is inversely proportional to the viscosity of the sugar water solution imbibed (electronic supplementary material, table S2, figure 2*b*). If we approximate flow as if the alimentary canals of ants were cylindrical pipes, we can use the Hagen–Poiseuille equation

$$1/Q\;=\;\frac{8\pi \eta L}{\Delta p\;A^{2}},$$

where *Q* is the drinking intake rate, *L* and *A* the length and cross-sectional area of the ant throats, η the viscosity of the solutions and Δp the pressure drop that the ants generate by suction [33]. Thus, high viscosity makes drinking more time-consuming and may cause the ants to switch toward grabbing behaviour. Indeed, drinking intake rate for 10CMC was slower than that for 10% sugar water (Wilcoxon test with Bonferroni correction, 10CMC versus 10%: *p* = 0.013) and comparable to drinking intake rate for 40% and 50% sugar water (Wilcoxon test with Bonferroni correction, 10CMC versus 40: *p* = 0.58; 10CMC versus 50: *p* = 0.37). Drinking intake rate for 10CMC falls onto the linear adjustment for drinking intake rate for solutions without additives (figure 2*b*).

**Figure 3.**
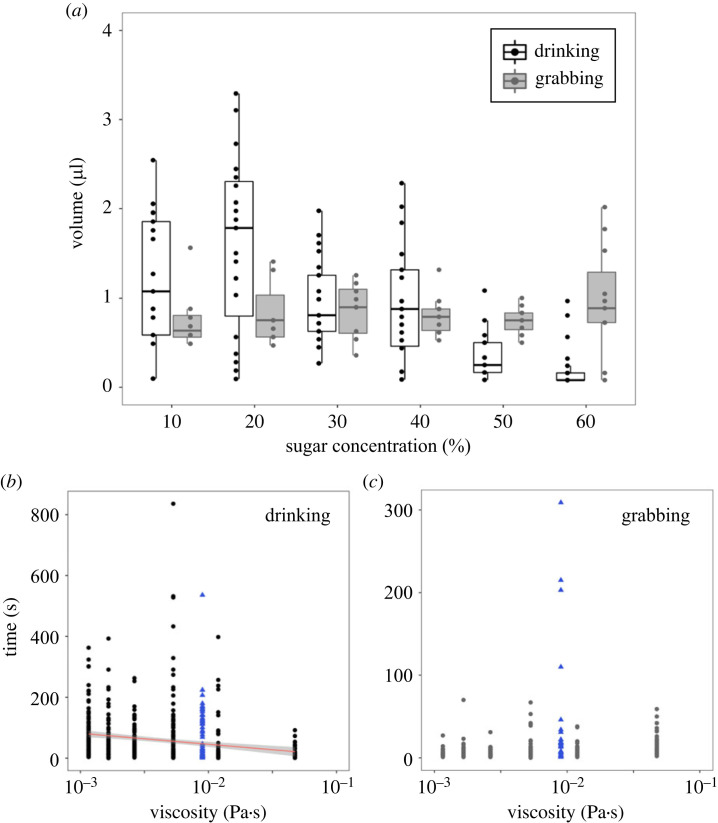
Impact of sugar concentration and viscosity on liquid collection. (*a*) The amount of liquid collected by drinking (black) and grabbing (grey) at different concentrations of sugar water (% w/w). Time spent drinking (*b*) and grabbing (*c*) by ants at different concentrations and viscosities of sugar water (% w/w). Six sugar concentration solutions are shown in black (*b*) and grey (*c*) circles, and the viscosity-altered solution 10CMC is shown with blue triangles. Statistical analysis can be found in table 2.

We investigated the volume of liquid food collected by ants using two different collection methods, feeding on different sugar concentrations (figure 3). For the volume ants collected through drinking or mandibular grabbing, we observed an interaction between sugar concentration and foraging action (figure 3*a*, table 2*a*, GLM, sugar × collection method: *p* < 0.001), and therefore we analysed the foraging actions separately. When ants drank liquid, the amount imbibed decreased as viscosity increased (figure 3*b*, table 2*b*, linear regression model (LM): *p* < 0.001). For the amount of liquid grabbed within the mandibles, there was no significant trend across the different viscosity (figure 3*c*, table 2*b*, LM: *p* = 0.14).

**Table 2.** Influence of sugar concentration, viscosity and foraging action on volume and handling time. Results of generalized and general linear regression (a) and linear regression model (b) between two explanatory variables and volume of carried or drunk water and handling time. Sugar concentration water is 10, 20, 30, 40, 50 and 60% (w/w) and foraging actions are drinking (DR) and mandibular grabbing (GR). Viscosity is seven different sugar and viscosity solutions; 10, 20, 30, 40, 50, 60% (w/w) and the viscosity-altered solution 10CMC.

(a) generalized linear regression						
	explanatory variables	estimate	std. error	*p*-value		
volume	sugar concentration	−0.026	0.003	<0.001		
	foraging actions (DR or GR)	−1.05	0.21	<0.001		
	sugar * foraging actions	0.028	0.0054	<0.001		
handling time	viscosity	−1325.942	193.878	<0.001		
	foraging actions (DR or GR)	−62.635	6.594	<0.001		
	viscosity * foraging actions	1386.844	310.709	< 0.001		
(b) linear regression						
		explanatory variables	estimate	std. error	*t*-value	*p*-value
volume	drinking	(intercept)	1.81	0.13	3.6	0.001
		sugar concentration	−0.026	0.0034	−7.57	0.001
	grabbing	(intercept)	0.76	0.11	7.19	<0.001
		sugar concentration	0.002	0.0027	0.75	0.41
handling time	drinking	(intercept)	75.4933	4.4055	17.136	<0.001
		viscosity	−1.3259	0.2322	−5.709	<0.001
	grabbing	(intercept)	9.609	3.263	2.944	<0.01
		viscosity	2.069	1.403	1.475	0.14

Since the volumes of droplets collected by mandibular grabbing were more or less constant (approx. 1 µl) at all sugar concentrations (figure 3*a*), the energy required to extract the droplets should be solely a function of the surface tension, where it should be easier to grab lower sugar concentration solutions due to the lower surface tension (table 1). Our measurements showed that the increase in surface tension with the addition of sugar is balanced by the increase in density, resulting in capillary length remaining unchanged (table 1). These values lead to an estimate of the maximum transportable volume as a drop of approximately 80 ± 10 µl, estimated from the capillary length translated into the volume of a sphere. This volume is more than one order of magnitude above the maximum volume measured in this study (table 1, figure 3*a*). Thus, at all sugar concentrations, the droplets transported by grabbing could sustain their own shape, were not impacted by gravity and did not flow during transport. This suggests that the limiting factor of the grabbing volume is not physical liquid properties, but more likely the ants’ morphological constraints.

Regarding the collection time for drinking or mandibular grabbing, we found an interaction between viscosity and foraging action (table 2*a*, GLM, viscosity × foraging action: *p* < 0.001). When ants drank, the drinking time decreased with increasing viscosity (figure 3*b*, table 2*b*, LM: *p* < 0.001). There was no linear relationship between grabbing time and viscosity (figure 3*c*, table 2*b*, LM: *p* = 0.14). All in all, mandibular grabbing took less time to collect liquid when compared to drinking. Thus, mandibular grabbing is a more effective method to collect liquid food, in terms of volume per foraging time, across different sugar concentrations.

**Figure 4.**
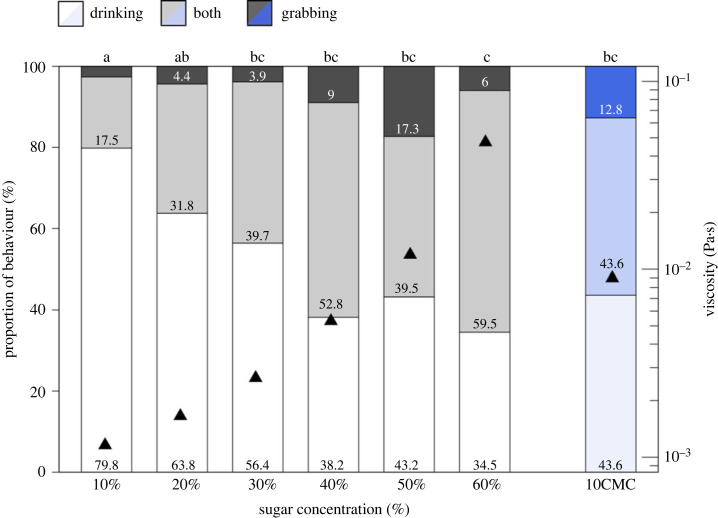
The proportion of pseudotrophallaxis depends on sugar concentration and viscosity. Drinking (white), both (grey) and grabbing (dark grey) indicate the behaviour of only pseudotrophallaxis, pseudotrophallaxis after drinking and only drinking, respectively. The blue bars relate to the viscosity-altered solution 10CMC. Differing letters on the top of the bars indicate significant differences at *p* < 0.05 (χ^2^ test with Bonferroni correction).

In spite of this, ants often collected sugar water in their mandibles after drinking and rarely performed only mandibular grabbing without drinking (figure 4). The proportion of these foraging actions was significantly different across sugar concentrations (figure 4, *p* < 0.05, χ^2^ test with Bonferroni correction). The proportion of mandibular grabbing after drinking (both) and mandibular grabbing alone (both of which result in pseudotrophallaxis) increased with increasing sugar concentration. This indicates that ants switch to grabbing and pseudotrophallaxis when they feed on liquid food with higher concentrations of sugar. This could come about because this high-sugar food is more valuable or because high-viscosity liquids are difficult for them to drink, as indicated by the drinking intake rate measurements (figure 2). To test whether ants react to changes in sugar concentration or viscosity, we offered ants our viscosity-altered solution 10CMC and observed which foraging action was used. The proportion of ants drinking significantly decreased compared to those drinking 10% sugar water (figure 4, *p* < 0.05, χ^2^ test with Bonferroni correction). The proportion of ants drinking 10CMC was equivalent to those drinking a high-viscosity 50% sugar solution (figure 4). This result shows that ants switch collection methods in response to viscosity, and not to sweetness.

**Figure 5.**
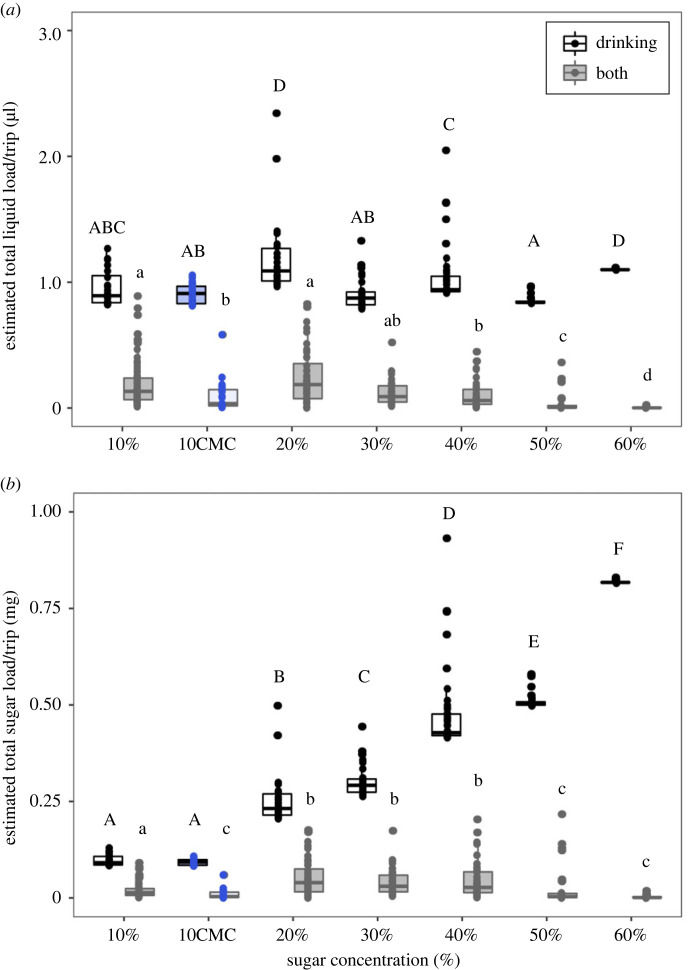
Foraging efficiency for the foraging action. Estimated total liquid (*a*) and sugar load (*b*) per trip are estimated based on drinking time and foraging action. Grey and white boxes indicate the actions of drinking and both drinking and grabbing, respectively. Blue indicates the 10CMC viscosity-altered solution. Statistical analysis can be found in table 3. Differing letters above points indicate significant differences of *p* < 0.05 (Wilcoxon test with Bonferroni correction).

To investigate whether this transition towards grabbing over drinking with increasing viscosity is related to foraging efficiency, we estimated the liquid load and sugar load per trip across the different sugar concentrations. The liquid load per trip had the largest volume when ants used both drinking and grabbing in the same trip (figure 5*a*). There was a significant interaction between sugar concentration and foraging action on the liquid load (table 3, ANOVA, sugar × foraging action: *p* < 0.01). The total liquid load when grabbing was larger than the crop load (table 3, ANOVA, foraging action: *p* < 0.05). The crop load decreased with increasing sugar concentration (Tukey's HSD Test, *p* < 0.001). To examine how much energy ants can bring back to the nest through these methods, we transformed the liquid load to sugar load. There was a significant interaction between sugar concentration and foraging action (table 3, ANOVA, sugar × foraging action: *p* < 0.01). The total sugar load acquired by grabbing significantly increased with sugar concentration (Wilcoxon test with Bonferroni correction, *p* < 0.05). The sugar loads collected by drinking were higher in the 20–40% of sugar concentrations (figure 5*b*, Wilcoxon test with Bonferroni correction, *p* < 0.05). Compared to the solution of similar viscosity (40% sugar water), the sugar intake by drinking of 10CMC was less per foraging trip (Wilcoxon test with Bonferroni correction, *p* < 0.05). In addition, for the high-viscosity and low sugar water 10CMC, the total sugar load per trip was lower than for the similar sugar concentration 10% sugar water (figure 5*b*, Wilcox test with Bonferroni correction, *p* < 0.05).

**Table 3.** Influence of different concentrations and viscosity of sugar water and foraging action on liquid and sugar load. Results of two-way ANOVA between two explanatory variables and liquid load or sugar load per trip. Solutions are seven different sugar solutions 10, 20, 30, 40, 50 and 60% (w/w) and 10CMC. Collection methods are drinking (DR) and both drinking and mandibular grabbing (both).

	explanatory variables	d.f.	sum Sq	mean Sq	*F* value	*p*-value
liquid load	solutions	6	8.66	1.44	60.12	<0.001
	collection methods (DR or both)	1	92.13	92.13	3838.72	<0.001
	solution * collection methods	6	1.55	0.26	10.74	<0.001
	residuals	515	12.36	0.02		
sugar load	solutions	6	13.758	2.293	1154.6	<0.001
	collection methods (DR or both)	1	14.902	14.902	7503.6	<0.001
	solution * collection methods	6	6.841	1.140	574.1	<0.001
	residuals	515	1.023	0.002		

To understand why ants might drink as well as grab, we investigated how often failures occur during mandibular transportation and whether carrying a mandibular droplet causes ants to walk more slowly. In our experimental set-up, which comprised a flat and relative short foraging path (approx. 30 cm), all ants succeeded in transportation by mandibular grabbing (electronic supplementary material, table S3). For both mean walking speed and maximum walking speed, there were no significant differences across the three categories: empty-crop, crop-full and crop- and mandible-full (electronic supplementary material, figure S3, Tukey-Kramer test). Even when crop-full ants held a droplet in their mandibles (ants with both full-crop and full-mandibles), their walking speed did not decrease compared to ants in empty-crop and full-crop conditions (electronic supplementary material, figure S3). These data suggest that grabbing is a more efficient method than drinking to collect high-viscosity liquid.

## 3. Discussion

Behaviour in a given species is highly adapted to that organism's context, and these behavioural adaptations often involve precise forms of behavioural plasticity. In this study, we analysed the flexibility of foraging behaviours in response to viscosity in *Diacamma* cf. *indicum*. The ant used two liquid collection actions – drinking and grabbing – when collecting liquid food. In this study, we aimed to quantify dynamic switching between two types of liquid food collection used by a single ant species. *Diacamma* cf. *indicum* is a part of a clade of ants that rarely specialise on liquid food. Given this species' phylogenetic context, these behaviours are likely to be relatively recent specialisations [15]. However, it remains unclear when ants use pseudotrophallaxis as opposed to true trophallaxis to collect, transport and share liquids, whether the use of these behaviours varies according to food quality, and whether one is an evolutionary step towards another.

### (a) Viscosity dictates behaviour

Here, we clearly observed mandibular gabbing and pseudotrophallaxis in the laboratory in *Diacamma* cf. *indicum* and saw that their use of this collection behaviour changes with sugar concentration and viscosity. Our results are consistent with previous studies in other ponerine ants, where ants stopped drinking [34] and tended to use mandibular grabbing at higher sugar concentrations (greater than 40%) [35]. By decorrelating viscosity from sugar content, our work revealed that ants made this switch in collection mode according to viscosity only (figure 4). Viscosity has been seen to reduce the liquid intake rates in many insects, including ants [25,34,36–38], consistent with our results (electronic supplementary material, figure S2). Here, we directly measured the drinking intake rate of the ants. We show a robust linear relationship between the inverse of drinking intake rate and the viscosity of the solutions regardless of the presence of the viscosity-altering agent, indicating that viscosity is the main factor regulating drinking time. Our analyses of surface tension and capillary length indicate that the biophysical limits for the maximum droplet volume of these solutions that *Diacamma* ants could transport are never reached. Rather, the constraints on volume obtained by grabbing are more likely related to the ants’ size and mandible span, which could be explored in future studies for example in wasps, which are physically larger and also transport fluids between their mandibles.

We found that ants used mandibular grabbing after drinking liquid (figure 3*a*). This maximizes liquid load per trip because ants can transport both internally and externally. Multiple trips may be costly as they involve loss of energy and increased predation risk, not only for the forager herself, but also for the colony as a whole. Foragers moving in and out of the nest reveal the location of the nest entrance to predators, putting the brood and reproductive individual at risk.

We observed no foraging failures or reduced travel speeds when ants were internally and externally loaded. Thus, the key factor in evaluating foraging efficiency would be calorie intake per trip. The total sugar load clearly increased at higher sugar concentrations when ants used mandibular grabbing. If ants were optimizing sugar returned to the nest per unit time while collecting (as opposed to per trip), grabbing only would be the optimal collection behaviour across viscosities. This suggests a colony-level optimization, like the optimization that minimizes energy consumption per transport energy that is seen in studies of foraging honeybees [39,40]. Future studies changing the length of the trip between the nest and the food resources or altering predation risk could further elucidate the reason for optimization per trip.

One possibility is that ants drink to fulfill individual energetic needs and transport liquid for the community by grabbing. The sugar load by drinking 10% sugar water was greater than the high-viscosity 10CMC solution (figure 5). This might be caused by the fact that ants can drink less high-viscosity fluid per unit time than low-viscosity fluid. If the volume of liquid imbibed was solely taken to cover individual energetic foraging costs then they would drink the same sugar content, which was not the case here.

### (b) Why do they not always use both drinking and mandibular grabbing?

One hypothesis could be that mandibular grabbing is a risky, but high pay-off collection method. For example, if ants encounter predators, they might not react quickly enough, ending up losing their mandibular droplet and/or being preyed upon because they are less agile. Ants might not use mandibular grabbing in dangerous sites where they encounter predators. On the contrary, if there are competitors around the food site, ants need to compete against other ant species. *Pheidole megacephala* soldiers reacted to the presence of competitors. The soldiers performed more mandibular grabbing on the territory of other ant species in order to rapidly gather and transport large loads of liquid food [41]. Future studies analysing transport time over different distances and in different ecological contexts could help elucidate the cost of transportation through pseudotrophallaxis.

In addition, the speed of sharing with nest-mates may differ between trophallaxis and pseudotrophallaxis. After foragers return to the nest, they share the liquid food through trophallaxis (regurgitation), or through pseudotrophallaxis. Trophallaxis should more rapidly distribute food in the colony because a receiver can become a donor and continue to distribute liquid food by regurgitation, also allowing the formation of a more complex social network [42–44]. In the carpenter ant *Camponotus*, foragers give food to a receiver proportionally to the available capacity in the receiver's crop. This trophallactic interaction helps the forager to sense colony satiation level and decide when to leave the nest and bring in more food [45]. However, the distribution dynamics of liquid by pseudotrophallaxis have not been studied. Observation of liquid distribution in the focal species that use both trophallaxis and pseudotrophallaxis is needed to understand these dynamics of liquid distribution and the regulation of foraging effort.

### (c) Why do so few ant species perform mandibular grabbing?

Mandibular grabbing and pseudotrophallaxis are mostly performed by ponerine ants (Ponerinae), with only a few noted exceptions in other major ant subfamilies [38]. One possible reason why some ants use pseudotrophallaxis and others use true trophallaxis is that many ants have internal morphological adaptations for liquid intake, storage and regurgitation, such as an expandable gaster, an elastic crop, a larger mouth and throat and a highly developed proventriculus [7,15]. These adaptations possibly allow for greater flexibility regarding the intake of high-viscosity solutions [16,39]. Thus, species with these morphological adaptations may not need pseudotrophallaxis.

A second possibility is that there might be biophysical restrictions on whether an ant can collect a liquid drop between her mandibles. It is likely that small body size makes interactions with liquid droplets more dangerous due to the difficulty of extracting a liquid with a high surface tension [46]. We observed that ants make a ‘hasty’ motion at the end of the extraction of the droplet. The force needed to break the droplet away is also directly dictated by the surface tension of the liquid. For small ants, it may be more difficult to exert the force required to extract the droplet. Whether any relationship exists between the ability to perform pseudotrophallaxis and biophysical restrictions, related to body size, has not yet been studied, making this area well poised for study from a biomechanics perspective.

### (d) Sharing with nestmates: socially transferred materials

Trophallaxis allows for medium- to long-term food storage before redistribution, while pseudotrophallaxis does not. Thus, ecological contexts and environmental harshness may also influence an ant to engage in one behaviour or another. Another valuable feature of trophallaxis is that donors can alter the contents of what they pass to nest-mates, either through partial digestion or through more complex signalling [47–49], which may bias a species or even a single ant to use one or another behaviour. Recent studies reveal that ants' regurgitated fluid contained more than food [47,48]. For example, trophallactic fluid in carpenter ants contains hormones, nest-mate recognition cues, small RNAs and various proteins. In *Diacamma*, it is unclear whether foragers regurgitate the contents of their crop during pseudotrophallaxis, or if they only regurgitate when they do trophallaxis. Future studies could examine whether they add any endogenous materials during these behaviours.

## 4. Methods

### (a) Colony collection and colony keeping

Colonies of *Diacamma* cf. *indicum* from Japan were collected from Kenmin-no-mori (Onna) and Sueyoshi park (Naha), Okinawa, Japan. The colonies were kept in plastic artificial nests filled with moistened plaster (9 cm diameter × 1.5 cm height). Each colony contained a mated gemma-possessing female (i.e. functional queen or gamergate), 50–150 workers, and brood. Nests were maintained at 25°C under a 12 h/12 h light–dark regime (light phase: 0800–2000 h). Reared colonies were fed with chopped frozen crickets three times per week. Water and 10% sugar water were provided ad libitum. All behavioural experiments were conducted between 12:00–19:00 in well-lit conditions at 25% and 50–60% humidity.

### (b) Physical properties of the liquid food sources

#### (i) Dynamic viscosity measurement

We prepared six sugar solutions, 10, 20, 30, 40, 50, 60% w/w. The concentrations we used were within the range reported for natural nectar sources (extrafloral nectars: 4.7–76% w/w [31]). In addition, we used two viscosity-altered solutions using carboxymethylcellulose sodium salt (CMC) (medium viscosity, Sigma-Aldrich), 10% sugar solution with 0.25% CMC (10CMC) and 30% sugar solution with 0.25% CMC (30CMC). By adding this product, we increased the viscosity of the solution without changing its sugar concentration. We confirmed that the sugar concentration of CMC additive solution was not changed using a saccharimeter (Refractometer RBR32-ATC).

We measured these solutions’ dynamic viscosity in a commercial stress-controlled rheometer (ANTON PAAR MCR 300) with a plate–plate geometry of 5 cm diameter and 0.5 mm gap. The bottom plate was roughened by sandblasting to prevent slip artefacts and the temperature was fixed at 25°C by a Peltier hood. We applied constant shear rates, and stress was computed when the steady-state regime had been reached for each shear rate. The resulting stress versus shear rate experiments exhibited linear behaviour characteristic of a Newtonian fluid. This was expected for sucrose/water solutions since sucrose is a small molecule that dissolves well in water, although this was not obvious for the CMC polymer solutions [50]. The dynamic viscosity was directly read from the slope evaluated by least-square minimization for each sample; for more detail see [51].

#### (ii) Surface tension and capillary length measurement

We measured the surface tension of six different sugar concentration solutions, 10, 20, 30, 40, 50, 60% w/w, with a commercial Du Noüy apparatus (Kibron EZPIPlus). The laboratory room was regulated at 25°C ± 1°C. Each measurement was repeated four times and the reported error is the standard deviation.

In order to obtain the capillary length, we used tabulated density measurements [52]. The capillary length is computed from surface tension and density through the following formula

$$\lambda_{c}=\;\sqrt{\frac{\gamma }{\rho g}}\;,$$

where γ is the surface tension, ρ the density and *g* the gravitational acceleration [53].

### (c) Features of foraging actions and choices

#### (i) Behaviour definitions

The ethogram of the social bucket method (encompassing mandibular grabbing, transport and pseudotrophallaxis) of *Diacamma* ant is shown in figure 1. Based on a previous study [24], two behaviours for liquid feeding and collection were defined: 1) drinking—individuals drink (i.e. mouthpart, labrum, attached to a liquid solution) and 2) grabbing—individuals open mandibles to grab and pull at a liquid solution, occasionally succeeding in collecting a droplet.

#### (ii) Drinking intake rate (volumetric flow rate, in µl s^−1^)

To investigate how ants' drinking intake rate changes depending on viscosity, we determined drinking intake rate. This was calculated as the slope (µl s^−1^) of the linear regression of crop load and corresponding drinking time as intake rate [54]. Also, the drinking intake rate allows us to infer drinking volume using the drinking time. We measured these two values (crop load and drinking time) for drinking events of 118 ants from six colonies, drinking seven different solutions: 10, 20, 30, 40, 50, 60% sugar w/w and 10CMC. The crop load (volume of sugar water drunk) was estimated as the difference in body mass before and after drinking (using a Fisherbrand analytical balance, to 0.1 mg). We chose ants outside of the nest and measured their body mass (before drinking). Then, each individual was separately placed into a plastic box (4.5 × 4.5 × 2 cm), containing a drop (approximately 100 µl) of sugar water. To measure the corresponding drinking time, we video-recorded the behaviour of ants when drinking using a digital camera (OLYMPUS STYLUS TG-3 Tough). Ants were offered one of seven different solutions: 10, 20, 30, 40, 50, 60% sugar w/w and 10CMC. When ants stopped drinking, we wiped the liquid off the mouthpart with a tissue and measured the ant's body mass to determine the volume of liquid food inside its crop. We estimated the volume (µl) of sugar water using the average weight (density) of the relevant sugar solution per 1 µl (electronic supplementary material, table S4). We weighed 100 µl droplets using a micropipette and an electronic balance (Semi-Micro Analytical Balances, GH-202, A&D Company).

We measured the time spent in contact with the droplet by manual observation as the corresponding drinking time. The drinking time was the total time during which the ant's mouthpart was attached to a liquid solution. We calculated the slope (µl s^−1^) of the linear regression as the drinking intake rate (electronic supplementary material, figure S1). Individuals were never tested more than once a day.

#### (iii) Measurement of volume of sugar water internally and externally carried

We measured the volume of water carried internally (crop load) and externally (mandible droplet), collected by 207 ants from 4 colonies. We measured the crop load, using the same weighing procedure as ‘drinking intake rate’ without the corresponding time measurements, which require maintaining filming and individual identity access measures. Ants were offered one of six different sugar concentrations: 10, 20, 30, 40, 50, 60% w/w. We did not measure the corresponding drinking time due to limitations in the setup.

Colonies were starved for 3–4 h before experiments (starvation time based on preliminary behavioural observations, data not shown). The volume of sugar water carried by mandibular grabbing was measured with a microcapillary tube. We placed an artificial nest on one side and a plate-shaped feeder (40 × 40 mm) on the other side of the foraging arena (460 × 260 × 100 mm; electronic supplementary material, figure S3). Ants were offered one of six different sugar concentrations: 10, 20, 30, 40, 50, 60% w/w. The ants freely accessed the offered food. After an ant succeeded in grabbing a droplet of the solution, we collected the droplet with a microcapillary tube during a return trip to the nest. The weight of the dispensed volume was measured to calculate the volume of sugar water the ant carried. We estimated the volume (µl) of sugar water using the average weight of the relevant sugar water (electronic supplementary material, table S4).

#### (iv) Grabbing time, drinking time and foraging actions in the foraging arena

We measured accumulated grabbing/drinking time during 572 foraging events from nine colonies. To test the effects of sweetness and viscosity on the foraging methods used, we used seven different sugar concentrations: 10, 20, 30, 40, 50, 60% w/w and 10CMC as a viscosity-altered solution. When foragers found 10CMC, they started tasting and drinking as usual. Refusal or avoidance-like behaviours were not observed towards 10CMC.

We placed an artificial nest on one side and a plate-shaped feeder (40 × 40 mm) on the other side of the foraging arena (460 × 260 × 100 mm; electronic supplementary material, figure S3). Ants were offered one of the seven sugar concentrations: 10, 20, 30, 40, 50, 60% w/w and 10CMC. We video-recorded the area around the sugar water droplet for 1 h using a digital camera (OLYMPUS STYLUS TG-3 Tough). We manually recorded one type of foraging action that foragers used with the droplet: ‘only drinking,’ ‘grabbing after drinking (both),’ or ‘only grabbing.’ Accumulated grabbing/drinking time for each foraging trip was calculated by an observer analysing the videos. The drinking time (s) is the accumulated time during which an ant's mouthparts were attached to the solution. We defined the start of grabbing as the time when the ant opened their mandibles because their mandibles are closed when this focal ant species drinks. The endpoint was when the ant succeeded in grabbing the droplet (e.g. detached droplet from solution). When ants repeated the grabbing, we included grabbing time until the last successful attempt.

### (d) Efficiency of foraging actions

#### (i) Estimation of sugar intake

To assess foraging efficiency, we estimated total sugar intake per foraging trip by combining several forms of data: 1) observation of foraging action ants performed; 2) drinking or grabbing time; 3) drinking intake rate; and 4) average grabbing volume. First, we calculated the total liquid load per trip based on the foraging action used (see §4b(iv)*, Grabbing time, drinking time and foraging actions in the foraging arena* and figure 4). The total crop load per trip was estimated by multiplying the drinking intake rate (see §4c(ii) Drinking intake rate (volumetric flow rate, in μl s^−1^), electronic supplementary material, figure S1) by the accumulated time drinking in the foraging arena (see §4b(iv) ‘*Grabbing time, drinking time and foraging actions in the foraging arena*’, figure 3*b*). The mandible load (collected by grabbing) was defined as the average volume of liquid carried for each sugar concentration in the experiment ‘*Measurement of volume of sugar water internally and externally carried*’ (see §4b(iii); figure 3*a*). When ants performed both drinking and grabbing, we summed the average volume of liquid carried and the estimated total crop load. Using the average weight of sugar water (electronic supplementary material, table S1), we converted the total liquid load per trip (µl) to the weight of the liquid load (mg). From the liquid load weight (mg), we calculated the total sugar intake per trip (mg) for each sugar concentration.

#### (ii) Walking speed and success rate of mandibular transport

We measured ants’ walking speed and success rate using each of the modes of liquid transport. We placed an artificial nest on one side and a plate-shaped feeder (40 × 40 mm) on the other side of the foraging arena (387 × 267 × 65 mm). Ants were offered one of the two sugar concentrations: 10% w/w (low sugar/low viscosity) and 50% w/w (high sugar/high viscosity). We continuously recorded the foraging arena for 1 h. The images were captured from above using a web camera (Logicool; HD Pro Webcam C920t) at 10 frames per second. We automatically extracted the coordinates of each individual using the video-tracking system, UMATracker [55]. We categorised ants' behaviours into 1) empty walking speed, 2) crop-full walking speed and 3) crop- and mandible-full walking speed by visual observation. We obtained walking speeds for 80 foraging trips from four colonies.

To investigate the success rate of mandibular liquid transport, we observed 30 foraging trips from the feeder to the nest from three colonies and investigated whether ants lost the mandibular droplet during the return trip. If an ant entered the nest without dropping the mandibular droplet, we recorded that as a foraging success. Returning to the nest after drinking was always considered a foraging success as the liquid cannot be lost.

### (e) Statistical analysis

Linear regression models were used to calculate the drinking intake rate. We compared regression slopes using a Wilcoxon test with Bonferroni correction. Generalized linear regression models were used to investigate the relationship between the liquid volume or loading speed with the food quality variables and foraging actions. Pairwise χ^2^ tests with a Bonferroni correction were used for comparing foraging action on different sugar concentrations. The relationship of the load with the food quality variables and foraging actions was analysed by two-way analysis of variance (ANOVA), followed by Tukey's honestly significant difference (HSD) test for multiple comparison of means. A significance level of 5% was used in all comparisons. All analyses were run in R studio 2022.02.3 (package: ggplot2), except for viscosity measurements and drinking intake rate that were analysed in python 3.9 and the linear regressions were done with the module scipy.optimize v1.7.1. [56].

## Data Availability

All data and codes are available at: https://doi.org/10.5061/dryad.x95x69png [57]. The data are provided in electronic supplementary material [58].
